# Association between night shift work and cardiovascular disease: a systematic review and dose-response meta-analysis

**DOI:** 10.3389/fpubh.2025.1668848

**Published:** 2025-09-24

**Authors:** Jiayu Xi, Wenlian Ma, Yanmin Tao, Xiameng Zhang, Linfeng Liu, Hongyan Wang

**Affiliations:** ^1^School of Basic Medical Sciences & School of Nursing, Chengdu University, Chengdu, China; ^2^Sichuan Provincial Orthopedic Hospital, Chengdu, China; ^3^School of Nursing, Sun Yat-sen University, Guangzhou, China; ^4^Chengdu University Affiliated Hospital, Chengdu, China; ^5^Sichuan Nursing Vocational College, Chengdu, China

**Keywords:** night shift work, cardiovascular disease, mortality, systematic review, meta-analysis

## Abstract

**Background:**

Shift work, particularly night shift work, has become increasingly prevalent on a global scale and is associated with multiple health issues including type 2 diabetes and cardiovascular diseases (CVD). This study aimed to assess the relationship between night shift work and the incidence and mortality of CVD.

**Method:**

Six electronic databases including PubMed, Embase, Cochrane Library, Web of Science, CINAHL, and Scopus were searched from inception until August 10, 2025. Cohort studies eligible for inclusion addressed the association between night shift work and outcomes of CVD. STATA 18.0 software was used for meta-analysis. The dose-response relationship was estimated using generalized least squares regression, and restricted cubic splines were used to analyze potential linear or nonlinear associations. The Newcastle-Ottawa Scale (NOS) was used to assess the quality of the studies. Quality assessment and data extraction were performed independently by two researchers.

**Results:**

Twenty-three cohort studies were included. Overall, this meta-analysis revealed that night shift work significantly increased the risk of total CVD events (RR = 1.13, 95% CI = 1.10–1.16) and total CVD mortality (RR = 1.27, 95%CI = 1.18–1.36). Dose-response analysis indicated that each 5-year increment in shift work duration was associated with a 7% higher risk of CVD incidence (RR = 1.07, 95% CI: 1.04–1.09) and a 4% increased risk of CVD mortality (RR = 1.05, 95% CI: 1.03–1.06). Subgroup analyses identified elevated risks for incident coronary heart disease (CHD) (RR = 1.22, 95% CI = 1.16–1.28) and ischemic heart disease (IHD) (RR = 1.09, 95% CI = 1.05–1.14), but not stroke (RR = 1.06, 95% CI = 0.95–1.18), and night shift work was associated with an increased risk of mortality due to CHD (RR = 1.22, 95% CI = 1.10–1.36), IHD (RR = 1.39, 95% CI = 1.06–1.84), and stroke (RR = 1.49, 95% CI = 1.04–2.12).

**Conclusion:**

These findings indicate that night shift work is significantly associated with increased CVD incidence and mortality risk, highlighting the need for targeted prevention strategies.

**Systematic review registration:**

https://www.crd.york.ac.uk/PROSPERO/view/CRD420251060086. CRD: 420251060086.

## Introduction

Contemporary socio-economic demands have led to the increasing prevalence of night shift work systems, particularly in the healthcare sector, service industries, and manufacturing industries (1). Studies indicate that approximately 18–20% of workers in Europe and the United States engage in shift work, encompassing various forms such as day shifts, night shifts, rotating shifts, and irregular schedules (2, 3). Shift work, commonly defined as work schedules not aligned with daytime hours, represents a widespread occupational arrangement globally (4). The shift toward such work patterns (especially the rise in night shifts) constitutes an independent risk factor for multisystem diseases by disrupting the body’s normal circadian rhythm.

Substantial evidence indicates that long-term night shift work is significantly associated with adverse health outcomes, including breast cancer (5), type 2 diabetes (6), mood disorders (7), and metabolic syndrome (8), and also contributes to the development and progression of cardiovascular diseases (CVD). CVD remains the leading cause of global mortality and a major contributor to the global disease burden (9). According to the World Heart Federation 2023 report, CVD caused approximately 20.5 million deaths globally, accounting for one-third of all global deaths (10). Among these CVD fatalities, 85% were attributable to heart attacks and strokes (11).

Accumulating evidence has linked prolonged night shift work to increased risks of CVD incidence and mortality (12–14). Although previous meta-analyses have reported associations between shift work and adverse CVD outcomes (15–18), one of these studies included only five cohort studies with limited sample sizes (15). More importantly, several recent cohort studies have since been published, providing substantial new evidence, however, some reported no significant association between night shift work and adverse CVD outcomes (14, 19–22). Given the current evidence remains inconclusive, an updated meta-analysis incorporating recent high-quality studies is imperative to clarify the potential impact of night shift exposure on cardiovascular disease morbidity and mortality.

Understanding the relationship between night shift work and CVD incidence and mortality is essential for establishing evidence-based occupational health standards. This study aims to systematically assess this relationship and synthesize evidence to provide clearer conclusions, ultimately informing interventions to mitigate night shift-associated CVD.

## Methods

### Protocol

The protocol for this systematic review and meta-analysis was registered to PROSPERO (CRD420251060086). This article followed the Preferred Reporting Items for Systematic Reviews and Meta-Analyses (PRISMA) guidelines (23).

### Search strategy

We comprehensively searched six electronic databases, including PubMed, Embase, Cochrane Library, Web of Science, CINAHL, and Scopus, conducted from inception to August 10, 2025, without language restrictions. The search strategy entailed a combination of exposure-related factors (“shift work,” “night work,” “Shift Work Schedule,” “night shift work,” “rotating shift”) and a mixture of Medical Subject Headings (MESH) terms and keywords pertinent to outcomes (“Cardiovascular Diseases,” “coronary heart disease,” “Myocardial Ischemia,” “ischemic heart disease,” “stroke,” “cardiovascular events,” “cardiovascular mortality”) (Detailed search strategy can be found in the Supplementary Table S1). Additionally, a manual search of the reference lists from the retrieved articles was conducted to identify any additional relevant studies.

### Eligibility

Inclusion criteria (based on PICO framework): P (Population): The studies examined outcomes in populations engaged in night shift work (including shift work schedules involving night shifts); I (Intervention): Any type of night shift work as the exposure variable (rotating shift work, fixed night shift work); C (comparisons): Daytime work exclusively, with no history of night shift work; O (Outcome): The outcomes focused on cardiovascular events (including both incidence and mortality) and encompassed major CVD subtypes such as coronary heart disease (CHD), ischemic heart disease (IHD), and stroke, and reported the provision of the 95% confidence intervals (CI), relative risk (RR), odds ratios (OR), hazard ratios (HR). When multiple publications utilized the same cohort, we prioritized the study with the most comprehensive follow-up, complete outcome data, or superior methodological quality to avoid double-counting of participants.

Exclusion criteria included: (1) studies with self-reported cardiovascular outcomes or those reporting only cardiovascular markers; (2) non-human studies; (3) reviews, case reports, letters, or conference abstracts; (4) incomplete data or inability to access full-text articles; (5) duplicate publications; and (6) case reports, systematic reviews, or meta-analyses.

### Study selection

Literature management and screening were conducted using EndNote 20. The literature screening procedure was carried out in two phases. Initially, two authors (XJY and MWL) assessed the eligibility of studies by evaluating the titles and abstracts according to the predefined inclusion and exclusion criteria. The second phase entailed a thorough examination of the full texts to ascertain which studies qualified for inclusion in the meta-analysis. In cases of disagreement during the literature screening, the issues were deliberated with a third author (TYM) until a consensus was achieved.

### Data extraction

Data regarding results and study characteristics were extracted to tables by the first author (XJY) and checked for accuracy by the second author (MWL). Microsoft Excel was used to extract the required data, including lead author, year of publication, location by country, sample source, study design, age, sex, sample size, follow-up duration, definition of night shift work, type of night shift work, occupation, Outcome, outcome assessment method, adjusted effect estimates (OR, RR, and HR) with 95% CI and a list of adjusted factors.

### Assessment of study quality

Two authors (XJY and MWL) independently assessed the quality of the studies using the nine-star Newcastle–Ottawa Scale (NOS) (24), which evaluates three domains: selection (0–4 points), comparability (0–2 points), and outcomes (0–3 points). Total scores were categorized into three groups: 0–3 (fair), 4–6 (moderate), and 7–9 (good). Inter-rater agreement in study quality assessment was quantified using Cohen’s kappa coefficient. According to Cohen’s kappa coefficient, inter-rater agreement was categorized into three levels: coefficients ≤0.60 indicated poor agreement, values between 0.61 and 0.80 represented moderate agreement, and coefficients ≥0.81 signified good agreement (25). Any disagreements were resolved through consensus discussion with the third author (TYM).

### Statistical analyses

Analysis of statistics performed with STATA 18.0. The RR was considered as the common measure of the association between night shift work and CVD. The HRs were deemed equivalent to RRs because HRs were considered to approximate RRs (26, 27). The ORs were transformed into RRs using the formula $\mathrm{RR}=\frac{\text{OR}}{\left(1-P_{0})+(P_{0}\times \text{OR}\right)}$, where *P*_0_ indicates the incidence of the outcome of interest in the non-exposed group, and then synthesized with the RRs and the HRs into pooled RRs (28). For each included study, we extracted covariate-adjusted outcome measures. When multiple effect estimates were reported within a single study, these were first pooled at the study level prior to the overall meta-analysis. Heterogeneity across studies was evaluated using Cochran’s Q statistic test and *I^2^* statistic (29, 30). A random-effects model was applied if significant heterogeneity was detected (*p*-value <0.05 or *I^2^* > 50%); otherwise, a fixed-effects model was utilized (31). Sensitivity analysis was conducted by sequentially excluding individual studies to assess the robustness of the pooled results. At least three quantitative categories (involving the duration of night shift work) were reported in the included studies and were incorporated into the dose-response meta-analysis. The dose-response relationship model proposed by Greenland and Longnecker (32) was adopted to calculate the trend based on the estimated values of logRR in different shift work categories. According to this method, the distribution of cases and participants, as well as RR and 95% confidence intervals, were extracted for each shift work category. Additionally, we used restricted cubic splines with three knots set at the 10th, 50th, and 90th percentiles of the distribution to assess the potential curvilinear association between years of night shift work and the risk of CVD incidence and mortality. In addition, subgroup analyses were conducted to stratify the results on cardiovascular outcome type, sex, type of night shift work, and geographic region. Publication bias was examined through visual inspection of funnel plots and quantified using Egger’s test, if there was a large publication bias, correction is performed using trim-and-fill method (33, 34).

### Quality of evidence

We assessed the strength of evidence for each outcome using the Grading of Recommendations Assessment, Development, and Evaluation (GRADE) framework. The evaluation included the following domains: risk of bias, inconsistency, indirectness, imprecision, and other considerations. Evidence quality was categorized into four levels: Very Low, Low, Moderate, and High.

## Results

### Search results

**Figure 1:**
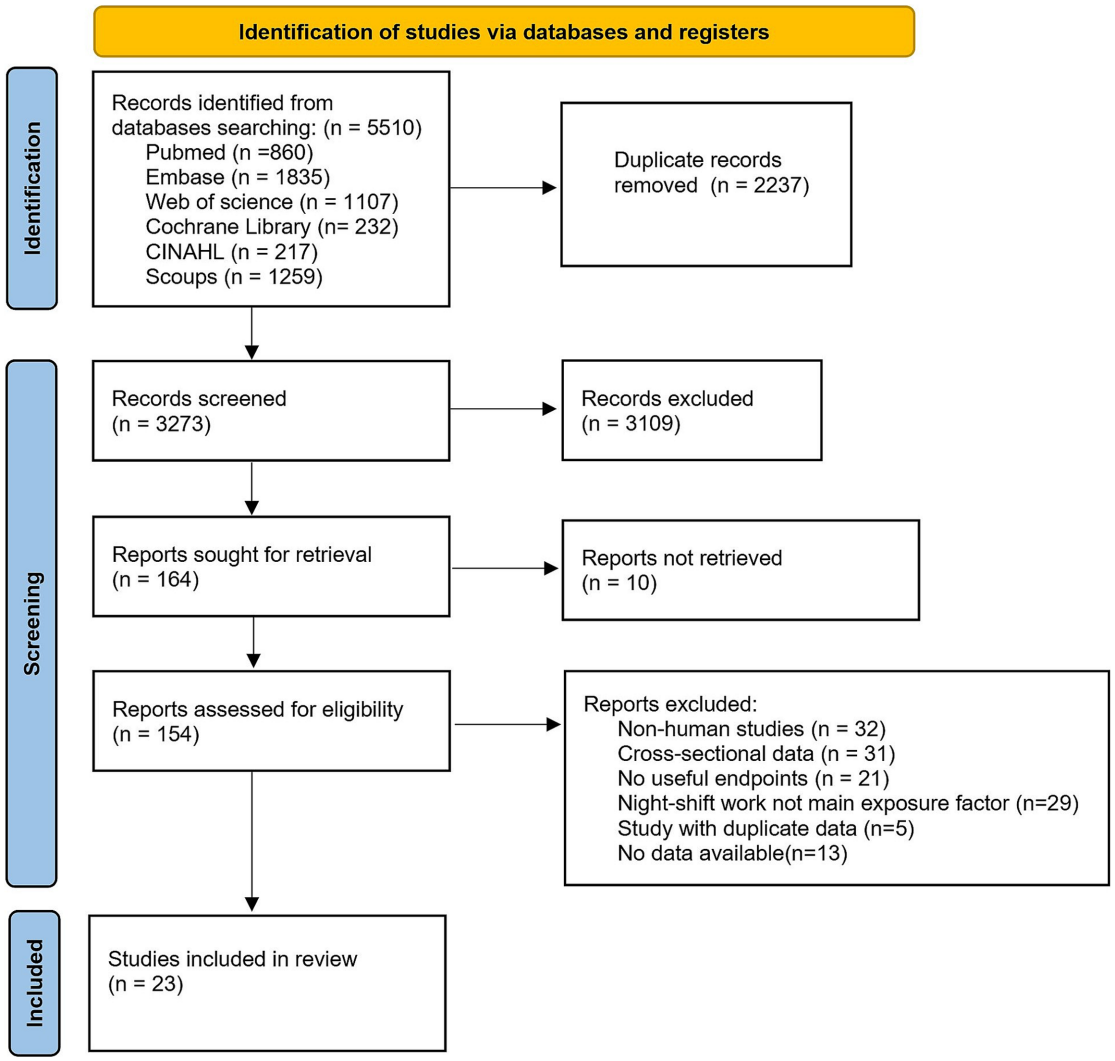
PRISMA flow diagram of the included studies.

The initial database search identified 5,510 articles: 860 from PubMed, 1,107 from Web of Science, 1835 from Embase, 232 from Cochrane Library, 217 from CINAHL, and 1,259 from Scopus. After removing 2,237 duplicates, 3,273 unique records underwent title and abstract screening. Of these, 154 articles were deemed eligible for full-text review. Following detailed evaluation, 131 studies were excluded based on predefined criteria: (1) non-human studies; (2) cross-sectional design; (3) irrelevant outcomes; (4) duplicate data from overlapping cohorts; (6) insufficient data availability. Ultimately, 23 articles met all inclusion criteria and were included in the meta-analysis (selection process detailed in Figure 1).

### Study characteristics

This meta-analysis incorporated 23 observational studies comprising 20 prospective cohort studies (13, 14, 19–22, 35–48), 3 retrospective cohort studies (49–51), with a total population of 3,340,377 participants. Geographically, 16 studies were conducted in Europe (13, 14, 19–22, 36, 37, 41–43, 45–47, 50, 51), 3 in North America (35, 38, 40), 1 in Oceania (48), and 3 in Asia (39, 44, 49). Regarding occupational distribution, 7 studies specifically focused on healthcare workers (19, 20, 22, 35, 38, 40, 42), while the remaining studies encompassed diverse occupations, predominantly involving industrial workers.

The included studies investigated associations between night shift work and CVD incidence/mortality risks, primarily targeting the following outcomes: CVD, CHD, IHD, and stroke. Case ascertainment was primarily based on local hospital admission records and death certificates. Definitions of night shift work varied across studies: most involved rotating night shifts, whereas others assessed fixed night shifts. Study characteristics are summarized in Table 1.

**Table 1:** Characteristics of the included cohort publications.

Study, Year, Country	Design	Sample Source	Age	Sex	Sample Size	Follow-up Duration (years)	Definition of night shift work	Type of night shift work	Occupation	Outcome	Case ascertainment	Relative risk (95% CI)	Factors Adjusted for in Analyses
Bigert et al., 2022, Sweden (19)	Prospective cohort study	healthcare employees in Stockholm	≤40; 41–50; >50	M&F	30,460	4.7	persons working various shift types including night shifts, or persons working night shifts only	Rotating work; Fixed night work	Healthcare workers	Stroke incidence	National patient registrations; the regional database	Rotating work: 1.37 (0.79 2.36); Fixed night work: 1.23 (0.68 2.25)	Sex, age, country of birth, level of education Occupation
Brown et al., 2009, US (35)	Prospective cohort study	Nurses health study	54.5 (7.2)	F	80,108	16	≥ 3 night shifts per month (including day and evening shifts)	Rotating work	Healthcare workers	Stroke incidence	Medical records; death certificates; imaging reports	1.11 (0.78 1.57)	Age, questionnaire cycle, hypertension, coronary heart disease, diabetes, elevated cholesterol, aspirin use, body mass index, smoking, alcohol consumption, fruit and vegetable consumption, physical activity, menopausal status and use of hormone replacement therapy
Bøggild et al., 1999, Denmark (36)	Prospective cohort study	Copenhagen male study	40–59	M	5,249	22	Shift work, night work or irregular working hours	Rotating work	Non-healthcare workers	IHD incidence	Hospital discharge records, National Health Service registrations and death certificates	1.00 (0.90 1.20)	Age, social class
Bunescu et al., 2024, Romania (37)	Prospective cohort study	a single Romanian automotive enterprise	37.78(9.17)	M&F	4,683	0.3	Working hours from 11 pm to 7 am	Fixed night work	Non-healthcare workers	CVD incidence	Medical records	M&F = 1.71 (1.09 2.63); *M* = 1.72 (1.07 2.76); *F* = 1.58 (0.46 5.42)	Age
Chang et al., 2025, UK (47)	Prospective cohort study	UK Biobank	37–73	M&F	283,579	14	Work schedule involving normal sleeping hours (12 am–6 am)	Rotating work	Non-healthcare workers	CVD mortality	Death certificates	2.08 (1.16 3.71)	Age, sex, race, Townsend deprivation index, education, BMI, smoking, alcohol, physical activity, diet, comorbidities, medications, family history, sleep duration, chronotype, job tenure
Vetter et al., 2016, US (38)	Prospective cohort study	Nurses health study 1; Nurses health study 2	NHS1:54.5; NHS2:34.8	F	NHS1:73,623; NHS2:115,535	24	≥ 3 night shifts per month (including day and evening shifts)	Rotating work	Healthcare workers	CHD incidence; CHD mortality	Medical records; death certificates	NHS1:1.18 (1.10 1.26); 1.29 (1.09 1.51); NHS2:1.27 (1.09 1.48); 1.09 (0.75 1.59)	Age, physical activity, diet, alcohol consumption and pack-years of smoking, parental history, menopausal status, parity, post-menopausal hormone use, multivitamin use, acetaminophen use, NSAIDs use and aspirin use, hypertension, hypercholesterolemia, diabetes, body mass index, race and husbands’ highest educational level
Eng et al., 2022, New Zealand (48)	Prospective cohort study	employed at the time of the 2013 census	20–64	M&F	1,594,677	5.8	≥ 3 h (12 am-5 am)	Rotating work	Non-healthcare workers	IHD incidence	Mortality records; hospital discharges	M = 1.10 (1.05 1.14) *F* = 1.25 (1.17 1.34)	Age, deprivation, smoking, ethnicity, other occupational exposures (noise, sedentary, long hours)
Ellingsen et al., 2007, Middle East (49)	Retrospective cohort study	Fertilizer plant workers	46.1 (7.0); 49.3 (6.7)	M	2,562	31	Rotating shifts, 2 mornings-2 afternoon- 2 nights system	Rotating work	Non-healthcare workers	CHD incidence	Medical records	1.62 (1.20 2.18)	Age at entry, job class, BMI, total cholesterol, triglycerides, smoking status, diabetes mellitus
Fujino et al., 2006, Japan (39)	Prospective cohort study	Population study	48.5 (5.9)	M	17,649	13.2	Fixed night or rotational shift	Rotating work; Fixed night work	Non-healthcare workers	IHD mortality	Death certificates	Rotating work:2.32 (1.37 3.95); Fixed night work: 1.23 (0.49 3.10)	Age, smoking, alcohol consumption, educational level, perceived stress, past medical history, body mass index, hours of walking, hours of exercise, and job type
Gu et al., 2015, US (40)	Prospective cohort study	Nurses health study	64.6 (7.1)	F	71,857	22	≥3 nights/month + day/evening shifts	Rotating work	Healthcare workers	CVD mortality	Medical records; death certificates	1.23 (1.09 1.38)	Age, alcohol consumption, physical exercise, multivitamin use, menopausal status, postmenopausal hormone use, physical exam in the past 2 years, healthy eating score, smoking status, pack-years, BMI, husbands’ education
Ho et al., 2021, UK (13)	Prospective cohort study	UK Biobank	52.43 (7.04)	M&F	238,661	11	Work at least 3 h between 11 pm and 6 am	Rotating work	Non-healthcare workers	CVD; IHD; Stroke incidence;	Death certificates and hospitalization records	1.11 (1.06 1.19); 1.09 (1.03 1.15); 1.09 (0.99 1.20)	Age, sex, education, deprivation additionally, hours of work per week, duration of current job, walking, standing at work, heavy manual/physical work additionally
Hublin et al., 2010, Finland (41)	Prospective cohort study	Twin study	40.2 (24–60)	M&F	20,142	22	Mainly shift-work	Rotating work	Non-healthcare workers	CHD mortality	Nationwide official registers	M = 1.09 (0.82 1.44); *F* = 1.22 (0.83 1.79)	Age
Jankowiak et al., 2024, Germany (21)	Prospective cohort study	Gutenberg Health Study	35–74	M&F	8,167	5	Any work during 23:00–05:00 h	Rotating work	Non-healthcare workers	CVD incidence	Medical records; death certificates	1.19 (0.67 2.12)	age, sex, occupational variables
Jorgensen et al., 2017, Denmark (42)	Prospective cohort study	Danish nurse cohort	50.2	F	18,015	17.6	Fixed night or rotational shift	Rotating work; Fixed night work	Healthcare workers	CVD; IHD; Stroke mortality	National register of causes of death	CVD (Rotating) = 1.24 (0.87 1.77); (Fixed) = 1.71 (1.09 2.69); IHD (Rotating) = 1.22 (0.61 2.41); (Fixed) = 2.30 (1.07 4.92); Stroke (Rotating) = 1.24 (0.70 2.22); (Fixed) = 1.98 (0.92 4.27)	Smoking, pack-years, leisure-time physical activity, BMI, alcohol consumption, diet, pre-existing diseases, self-reported health, stressful work environment, marital status, female reproductive factors
Kader et al., 2022, Sweden (20)	Prospective cohort study	Sample Source healthcare employees in Stockholm	≤40; 41–50; >50	M&F	30,398	8	Mainly shift-work	Rotating work; Fixed night work	Healthcare workers	IHD incidence	National patient registrations; the regional database	Rotating work = 0.93 (0.58 1.49); Fixed night work = 1.61 (1.06 2.43)	Age, sex, education, country of birth, profession, number of total night shifts per year
Karlsson et al., 2005, Sweden (43)	Prospective cohort study	pulp and paper industry	10–59	M	5,442	50	Shift type: three shifts (morning, midday, night) or five shifts	Rotating work	Non-healthcare workers	CHD; Stroke mortality	National register of causes of death	1.24 (1.04 1.49); 1.51 (0.87 2.63)	Age
Li et al., 2021, China (44)	Prospective cohort study	Dongfeng-Tongji Cohort	61.7 (7.9)	M&F	21,802	7.0	Any work schedule that involves irregular working hours as opposed to a regular daytime work schedule	Rotating work	Non-healthcare workers	CHD incidence	Medical insurance records, hospital records and death certificates	1.28 (1.08 1.51)	Age, sex, educational level, family history of coronary heart disease, job category, cigarette smoking, regular physical exercise, nighttime sleep quality, amount of time spent in bed each night, napping, body mass index, hypertension, hyperlipidemia, diabetes
Larsen et al., 2019, Denmark (45)	Prospective cohort study	Danish Labour Force Survey	21–59	M&F	145,861	14	Exposure to night work	Rotating work	Non-healthcare workers	IHD incidence	National patient registrations	1.08 (0.98 1.19)	Calendar time, time passed since start of follow-up, job in health care sector, age, sex, SES and weekly working hours
Tenkanen et al., 1997, Finland (46)	Prospective cohort study	Helsinki Heart Study	40–55	M	1,806	6	Shift work (two and three shifts)	Rotating work	Non-healthcare workers	CHD incidence	National patient registrations	1.40 (1.00 1.90)	Age, lifestyle, lipids, blood pressure
Vestergaard et al., 2023, Denmark (22)	Prospective cohort study	register-based nationwide cohort study	≥18	M&F	254,031	8	A day with ≥3 h of work between 12:00 am (midnight) and 5:00 am	Rotating work	Healthcare workers	CHD incidence	National patient registrations	*M* = 1.22 (1.07 1.39); *F* = 1.06 (0.97 1.17)	Age, diabetes, obesity, hypercholesterolaemia, hypertension, family history of cardiovascular disease, calendar year, occupation and educational level
Wang et al., 2021, UK (14)	Prospective cohort study	UK Biobank	51.3 (6.8)	M&F	283,657	10.4	Working hours outside the normal daytime working hours of 9:00 am. to 5:00 pm.	Fixed night work	Non-healthcare workers	CHD; Stroke incidence	Death registration, primary health care and hospitalization records	1.22 (1.11 1.35); 0.90 (0.73 1.12)	Age, sex, ethnicity, Townsend index, education, seven ideal cardiovascular health metrics
Yadegarfar & McNamee, 2007, UK (51)	Retrospective cohort study	Nuclear fuel workers	48.5 (13.2); 47.8 (13.4)	M	1,270	25.5	Two and three shifts, any direction/ system	Rotating work	Non-healthcare workers	IHD mortality	Death certificates	1.21 (0.95 1.50)	Categorized systolic blood pressure, diastolic blood pressure, body mass index, height, smoking and social class
Yong M et al., 2014, Germany (50)	Retrospective cohort study	Chemical workers	41.2 (11.1)	M	31,143	10	12 h-shifts (fast-forward-rotating)	Rotating work	Non-healthcare workers	IHD mortality	Death certificates	0.96 (0.48 1.89)	Age, smoking, alcohol consumption, work level and disease status

CVD, cardiovascular disease; CHD, coronary heart disease; IHD, ischemic heart disease; M, male; F, female; BMI, body mass index; SES, socioeconomic status.

### Quality assessment

Supplementary Table S2 shows the quality assessment of the included studies. All 23 studies were rated as high-quality based on the NOS, with scores ≥7. High methodological quality was primarily manifested in cohort selection, ascertainment of night shift work exposure, and assessment of CVD outcomes. Some potential biases were noted, primarily arising from inadequate follow-up and comparability limitations. Inter-rater reliability was assessed using Cohen’s kappa coefficient. The analysis yielded a kappa value of 0.857 (*p* < 0.001), indicating good agreement between the two assessors in quality evaluation.

### Association between night shift work and total CVD incidence

**Figure 2:**
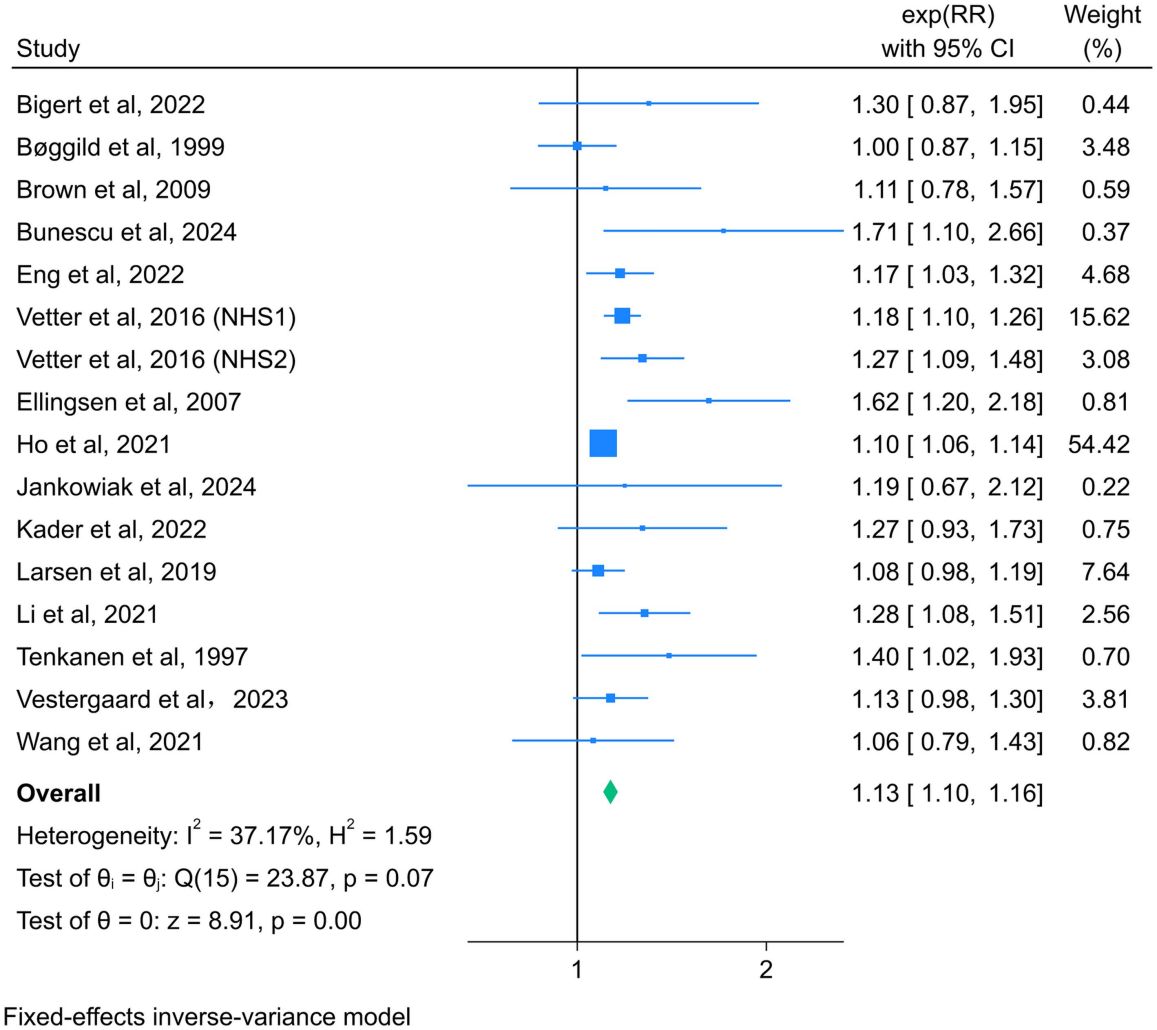
Forest plot of night shift work and total CVD incidence.

Fifteen studies reported the association between night shift work and the risk of total CVD events, with a total of 2,891,280 participants (13, 14, 19–22, 35–38, 44–46, 48, 49). Due to the absence of significant heterogeneity among the studies (*I^2^* = 37.17%, *p* = 0.07), a fixed-effect model was selected for analysis. The summary RR (95% CI) was 1.13 (1.10–1.16) (Figure 2), suggesting that night shift work was associated with a higher risk of adverse cardiovascular events compared to daytime work.

### Association between night shift work and total CVD mortality

**Figure 3:**
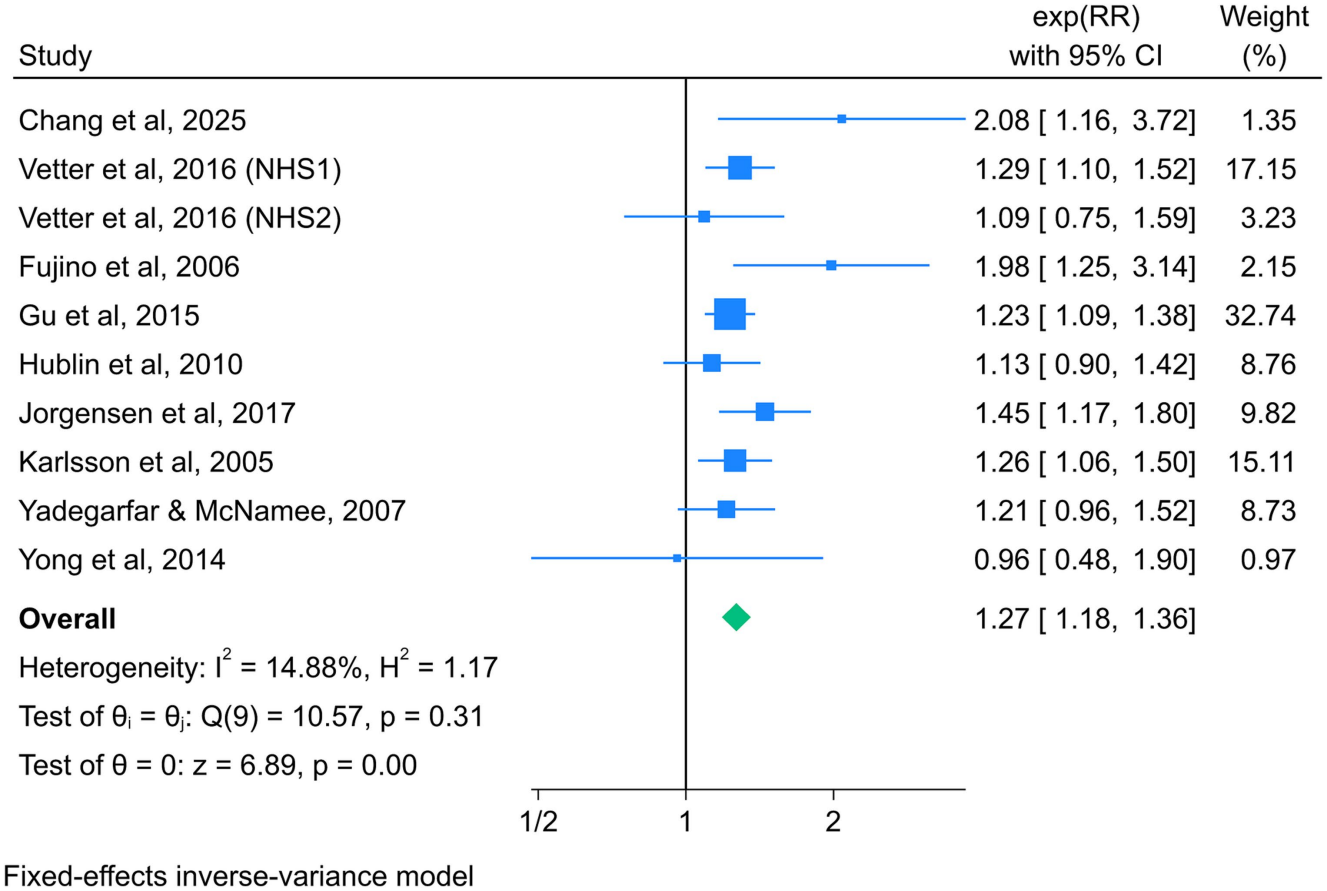
Forest plot of night shift work and total CVD mortality.

Nine studies reported the association between night shift work and the risk of total CVD mortality, with a total of 638,255 participants (38–43, 47, 50, 51). Given the absence of significant heterogeneity across studies (*I^2^* = 14.88%, *p* = 0.31), a fixed-effect model was selected for analysis. The summary RR (95% CI) was 1.27 (1.18–1.36) (Figure 3), suggesting that night shift work was associated with a higher risk of adverse cardiovascular mortality outcomes compared to daytime work.

### Dose response meta-analysis

**Figure 4:**
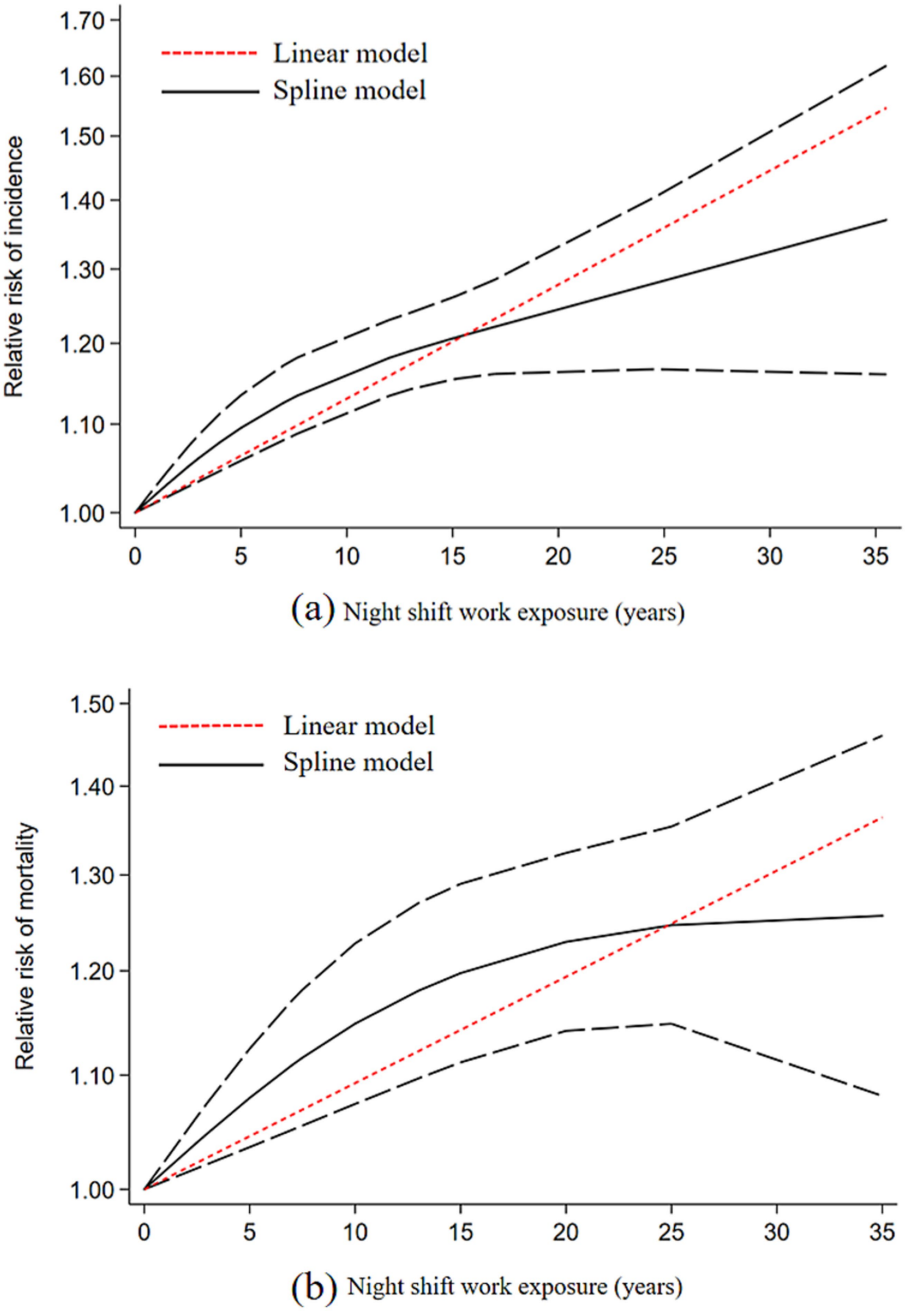
Dose-response relationship plot between duration of night shift work and CVD; **(a)** CVD incidence; **(b)** CVD mortality.

Studies with ≥3 categories of shift work duration were included in the dose-response analysis. Results demonstrated a linear dose-response relationship between night shift work and the risk of CVD incidence and mortality. For each 5-year increase in shift work duration, the risk of CVD incidence increased by 7% (RR = 1.07, 95% CI: 1.04–1.09), and the risk of CVD mortality increased by 4% (RR = 1.05, 95% CI: 1.03–1.06) (Figure 4a). Using restricted cubic splines, we did not detect a significant nonlinear dose-response association between shift work duration and the risk of CVD incidence (*p* = 0.068) or mortality (*p* = 0.096) (Figure 4b).

### Subgroup analysis

**Figure 5:**
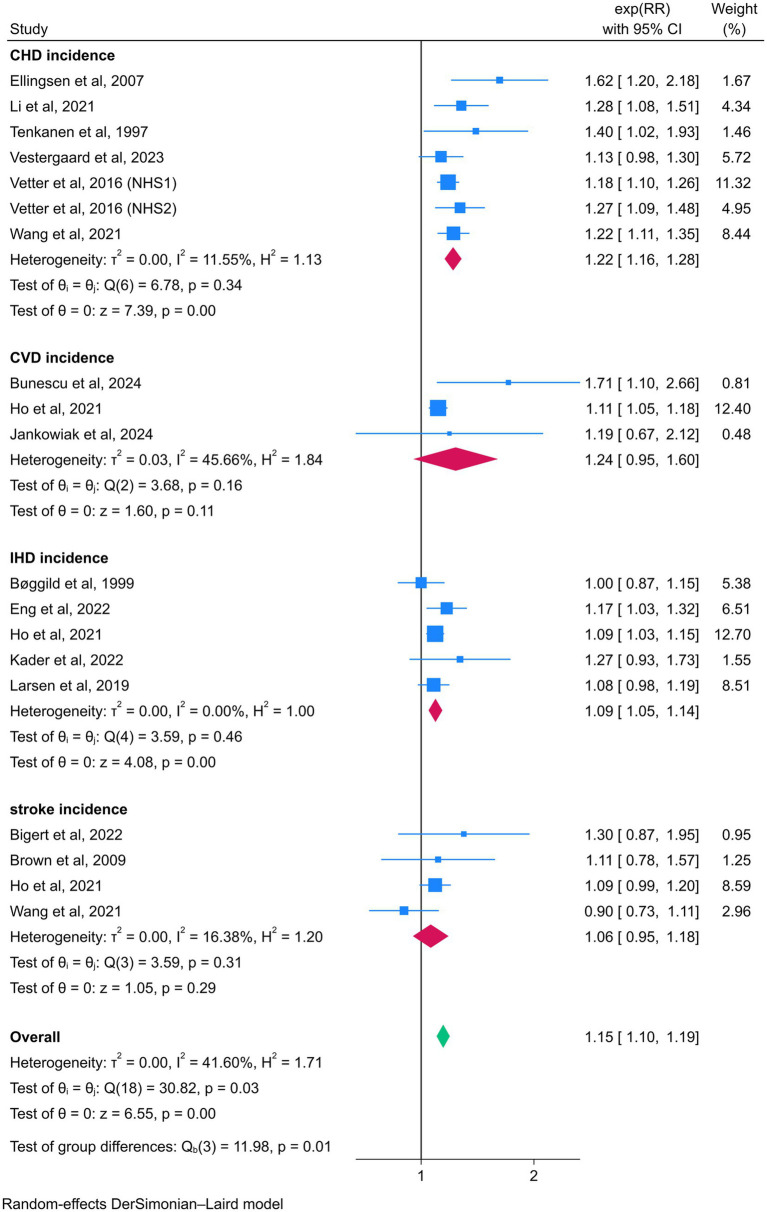
Forest plot of the associations between night shift work and CVD incidence by subgroup analysis.

**Figure 6:**
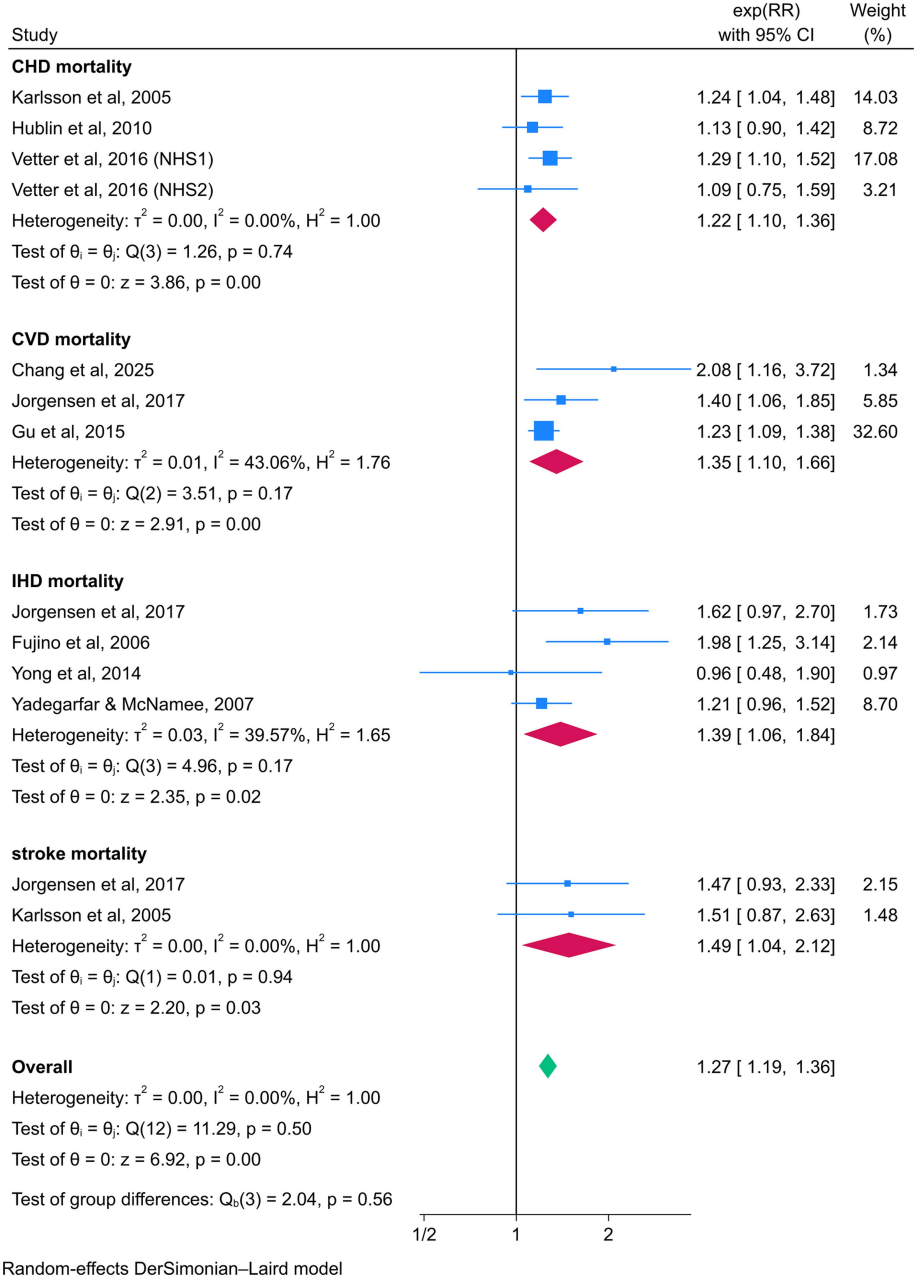
Forest plot of the associations between night shift work and CVD mortality by subgroup analysis.

In subgroup analyses examining the association between night shift work and CVD events, night shift workers exhibited significantly higher risks of CHD (RR = 1.22, 95% CI = 1.16–1.28) and IHD (RR = 1.09, 95% CI = 1.05–1.14) compared to daytime workers. However, no statistically significant associations were observed for CVD (RR = 1.24, 95% CI = 0.95–1.60) or stroke (RR = 1.06, 95% CI = 0.95–1.18). In contrast, subgroup analyses focusing on CVD mortality revealed elevated risks among night shift workers for CHD (RR = 1.22, 95% CI = 1.10–1.36), CVD (RR = 1.35, 95% CI = 1.10–1.66), IHD (RR = 1.39, 95% CI = 1.06–1.84), and stroke (RR = 1.49, 95% CI = 1.04–2.12) (Figures 5, 6).

Additionally, night shift work patterns were significantly associated with both cardiovascular event incidence and mortality: fixed night work (RR = 1.34, 95% CI = 1.04–1.72) and rotating work (RR = 1.15, 95% CI = 1.10–1.21) were positively correlated with CVD incidence, while fixed night work (RR = 1.77, 95% CI = 1.28–2.46) and rotating work (RR = 1.25, 95% CI = 1.16–1.35) were associated with increased CVD mortality. Sex-specific analyses indicated a 18% higher risk of CVD events in female (RR = 1.18, 95% CI = 1.11–1.26) and a 20% higher risk in male (RR = 1.20 95% CI = 1.07–1.36) among night shift workers. For CVD mortality, both male (RR = 1.27, 95% CI = 1.07–1.45) and female (RR = 1.27, 95% CI = 1.17–1.38) showed statistically significant increases in risk. Geographic region subgroup analyses further demonstrated that night shift workers from Asia, Europe, North America, and Oceania are all significantly associated with the incidence and mortality risk of CVD (Tables 2, 3).

**Table 2:** Subgroup analyses of the risk of total CVD incidence (type of night shift work, sex, geographic region).

Groups	Category	*N*	RR	95% CI	Heterogeneity (*I^2^*, %)
Type of night-shift work	Fixed	4	1.34	(1.04, 1.72)	31.12
	Rotating	14	1.15	(1.10, 1.21)	36.19
Sex	Male	6	1.20	(1.07, 1.36)	67.95
	Female	6	1.18	(1.11, 1.26)	44.19
Geographic region	Asia	2	1.39	(1.12, 1.74)	45.03
	Europe	10	1.10	(1.06, 1.15)	53.71
	North America	3	1.19	(1.12, 1.27)	0
	Oceania	1	1.17	(1.03, 1.32)	–

RR, Relative Risk; CI, confidence interval.

**Table 3:** Subgroup analyses of the risk of total CVD mortality (type of night shift work, sex, geographic region).

Groups	Category	*N*	RR	95% CI	Heterogeneity (*I^2^*, %)
Type of night-shift work	Fixed	2	1.77	(1.28, 2.46)	0
	Rotating	10	1.25	(1.17, 1.33)	0
Sex	Male	5	1.27	(1.08, 1.49)	25.15
	Female	5	1.27	(1.17, 1.38)	0
Geographic region	Asia	1	1.98	(1.25, 3.14)	–
	Europe	6	1.28	(1.14, 1.43)	17.33
	North America	3	1.24	(1.13, 1.36)	0

### Sensitivity analysis

In this study, a sensitivity analysis was conducted on the association between night shift work and the risk of CVD incidence (Supplementary Figure S1) and mortality (Supplementary Figure S3). The application of sensitivity analysis confirmed the reliability of our meta-analysis results.

### Publication bias

In this study, we used the Egger test and funnel plot to assess publication bias in the included studies. The analysis showed that the data distribution was slightly skewed in the association analysis between night shift work and overall CVD incidence risk (Supplementary Figure S2). The Egger test result (*p* = 0.025 < 0.05) indicated potential publication bias. However, the trim and fill method indicated that the impact of publication bias on the results was minimal. Imputation of six hypothetical studies showed no substantial alteration in the effect estimate (Supplementary Figure S2). This confirms the robustness of the primary conclusions. In the analysis of the association between night shift work and overall CVD mortality risk, the data distribution was generally symmetrical and relatively concentrated (Supplementary Figure S4). The Egger test results (*p* = 0.375 > 0.05) indicated no significant bias.

### Grading of the evidence

GRADE evaluation indicated predominantly low to very low certainty evidence (Supplementary Table S3). Given the observational cohort designs, initial certainty was low. Evidence for CVD incidence was downgraded to very low due to publication bias, while CVD mortality maintained low certainty absent downgrading factors. All other outcomes were rated very low, primarily because of imprecision and publication bias risks from limited studies.

## Discussion

This systematic review and meta-analysis of 23 cohort studies, encompassing data from over 3.3 million participants, provides a comprehensive evaluation of the association between night shift work and the risk of CVD incidence and mortality. Our results demonstrate that night shift work is significantly associated with an increased risk of both CVD incidence (RR = 1.13, 95% CI: 1.10–1.16) and CVD mortality (RR = 1.27, 95% CI: 1.18–1.36). Furthermore, dose-response analysis revealed a linear relationship between the duration of night shift work and CVD risk, with each additional 5-year period of exposure associated with a 7% increase in the risk of CVD incidence and a 4% increase in the risk of CVD mortality. These findings underscore a clear time-dependent effect of night shift work on cardiovascular health, providing crucial quantitative evidence for occupational health risk assessment.

Although our research findings are similar to some previous meta-analyses (15–18), there are still some differences. Regarding the risk of CVD incidence, our results are consistent with the conclusions of previous meta-analyses in this field. However, in terms of mortality risk, our study found a significant association, which is inconsistent with the results reported by Vyas et al. (16) and Torquati (2018) (16, 17). This difference may be related to the inclusion of more recent high-quality cohort studies in our research, with a larger sample size and higher statistical power, thereby identifying risk effects that may have been underestimated in previous studies. In terms of the dose-response relationship, our study supports the linear cumulative effect model reported by Wang et al. (15), indicating that risk accumulation begins from the start of night shift work (15). This is different from the non-linear model proposed by Torquati et al. (17), which suggests no significant risk in the first 5 years, and that meta-analysis only conducted dose-response analysis for CVD incidence. However, given the conflicting results from existing studies, there is a need for further longitudinal research to precisely quantify the exposure duration of night shift work in relation to cardiovascular health. In summary, in public health strategies and occupational health management, it is necessary to develop optimized shift cycle plans, avoid long-term consecutive night shifts, and reduce the risk of CVD.

Our findings indicate that the association between night shift work and CVD risk holds consistently across all subgroups, with the exception of stroke incidence. Notably, although stroke did not reach statistical significance in the risk of incidence, previous studies have suggested that male night shift workers may be more prone to stroke (52, 53). This difference may be related to gender differences in thrombosis tendency, hypertension management, or health behavior patterns. Additionally, in the subgroup analysis of stroke mortality risk, only two studies were included due to the limited number of studies, and the results should be interpreted with caution. Regarding shift types, we found that both rotating shift work and fixed night shift work were associated with the incidence and mortality risk of CVD, with fixed night shift work presenting a greater risk. This may be due to the fact that rotating shift work may include some non-night shifts as exposures, and the intensity of rotating shift work is lower than that of fixed night shift work (18). The gender subgroup analysis indicated that both men and women were associated with the incidence and mortality of CVD, with a more significant risk association in men. This gender difference may be attributed to men’s higher susceptibility to dyslipidemia, greater likelihood of engaging in physically demanding occupations, and higher prevalence of adverse lifestyle factors such as smoking and alcohol consumption (54). Notably, Torquati et al. previously did not find a significant association in men for the incidence of CVD (16). Geographically, the risk association was higher in Asian populations compared to Europe, North America, and Oceania. This regional difference may be related to variations in shift organization patterns and healthcare access among regions, but due to the limited data from Asia, this result should be interpreted with caution. Future studies could incorporate more detailed stratification based on sociodemographic factors, which is crucial for a comprehensive assessment of risk differences among populations.

Multiple mechanisms can account for the association between night shift work and the risks of CVD incidence and mortality. Night shift work is considered to be associated with circadian rhythm disruption, hormonal dysregulation, and stress responses, all of which are biological pathways that contribute to an increased incidence of CVD and mortality. Firstly, night shift work disrupts the synchronization between the endogenous circadian clock and the environmental light - dark cycle (55). This disruption impairs autonomic regulation and hormonal homeostasis, rendering individuals more susceptible to hypertension, insulin resistance, and metabolic dysfunctions (56). Furthermore, research has shown that night shift workers exhibit elevated levels of inflammatory biomarkers (57, 58). These include C-reactive protein, tumor necrosis factor-*α* (TNF-α), and white blood cell count. Such inflammatory responses accelerate the progression of atherosclerosis and elevate the risk of cardiovascular events. In addition, the high work stress associated with night shift work may trigger stress responses. This promotes the adrenal cortex to secrete glucocorticoids under the influence of adrenocorticotropic hormone, leading to obesity and insulin resistance (59, 60). With the accumulation of long-term exposure, this can further result in hypertension, hyperlipidemia, and other risk factors for cardiovascular disease (61, 62). Overall, the potential harm of night shift work to cardiovascular health is not single but multi-targeted.

A key strength of this study lies in the inclusion of a greater number of eligible studies than previous systematic reviews and meta-analyses on this topic, encompassing ten investigations not included in prior meta-analyses, which may support robust effect size estimates and increase the accuracy of meta-analysis. Furthermore, beyond examining overall CVD incidence and mortality risks, we specifically analyzed major CVD subtypes. Additionally, given the critical role of exposure duration in occupational health, we assessed both linear and nonlinear dose-response relationships between shift work duration and CVD incidence/mortality risks.

## Limitations and prospects

Several limitations should be acknowledged in this meta-analysis. First, heterogeneity in the definition of night shift work across studies may introduce measurement bias. Second, residual confounding from unmeasured factors (dietary habits, physical activity) cannot be fully excluded. Third, most included studies were conducted in European and North American populations, limiting generalizability to low-income regions. Fourth, reliance on self-reported questionnaires or interviews to assess night shift work in some studies, coupled with potential changes in shift patterns during follow-up, may result in misclassification or measurement errors. Additionally, the search strategy was limited to studies related to cardiovascular events, and the strict inclusion and exclusion criteria may have led to the exclusion of potentially relevant studies. Finally, some subgroups included only 1–2 studies, which may affect the generalizability of the results, and the analysis results should be interpreted with caution. Future studies should actively include prospective cohort designs from low- and middle-income regions to evaluate the generalizability of findings across diverse geographic settings. Furthermore, primary studies are encouraged to incorporate objective exposure assessment (electronic shift records) with repeated measurements to reduce confounding bias, and to more deeply explore the underlying biological mechanisms.

## Conclusion

In summary, this meta-analysis found an association between night shift workers and an increased risk of adverse cardiovascular outcomes. Further research into the underlying mechanisms and potential interactions between these factors is necessary in the future in order to develop targeted interventions and prevention strategies for individuals at risk of adverse cardiovascular outcomes due to shift work, while work organizations should try to scientifically and rationally design shift schedules to enable employees to be in a favorable occupational environment, in order to mitigate the risk of adverse cardiovascular outcomes.

## Data Availability

The original contributions presented in the study are included in the article/Supplementary material, further inquiries can be directed to the corresponding author.
