# Supplementary material for: Association between night shift work and cardiovascular disease: a systematic review and dose-response meta-analysis
Source: Front Public Health. 2025 Sep 24;13:1668848. doi: 10.3389/fpubh.2025.1668848 (PMC12506678; doi:10.3389/fpubh.2025.1668848)
Supplement: Supplementary file 1 [file Data_Sheet_1.docx]

Supplementary Material

# Supplementary Figures and Tables

## Supplementary Figures

### Supplementary Figure S1. Sensitivity analysis of the association between night shift work and total CVD incidence.

### Supplementary Figure S2. Publication bias plot of the association between night shift work and total CVD incidence.

### Supplementary Figure S3. Sensitivity analysis of the association between night shift work and total CVD mortality

### Supplementary Figure S4. Publication bias plot of the association between night shift work and total CVD mortality

### Supplementary Figure S5. Subgroup analysis of CVD incidence (type of night shift work, sex, geographic region)

A) Night shift work and CVD incidence: Meta-Analysis Stratified by type of night shift work

**B)** Night shift work and CVD incidence: Meta-Analysis Stratified by sex

**C)** Night shift work and CVD incidence: Meta-Analysis Stratified by geographic region

Supplementary Figure S6. Subgroup analysis of CVD mortality (type of night shift work, sex, geographic region).

A) Night shift work and CVD mortality: Meta-Analysis Stratified by type of night shift work

**B)** Night shift work and CVD mortality: Meta-Analysis Stratified by sex

**C)** Night shift work and CVD mortality: Meta-Analysis Stratified by geographic region

## Supplementary Tables

### Supplemental Table S1. Literature Search Strategy - Displaying Literature Search Terms

| Database | Step | Terms | Results |
| --- | --- | --- | --- |
| PubMed | 1 | ((((("Cardiovascular Diseases"[Mesh]) OR ("Coronary Artery Disease"[Mesh])) OR ("Myocardial Ischemia"[Mesh])) OR ("Coronary Disease"[Mesh])) OR ("Stroke"[Mesh])) OR (Cardiovascular Diseases[Title/Abstract] OR Coronary Disease[Title/Abstract] OR Coronary Artery Disease[Title/Abstract] OR Myocardial Infarction[Title/Abstract] OR ischemic heart disease[Title/Abstract] OR coronary heart disease[Title/Abstract] OR Stroke[Title/Abstract] OR cerebral infarction[Title/Abstract] OR cerebral hemorrhage[Title/Abstract] OR cardiovascular morbidity[Title/Abstract] OR cardiovascular mortality[Title/Abstract]) | 3222213 |
|  | 2 | ("Shift Work Schedule"[Mesh]) OR (shift work[Title/Abstract] OR night work[Title/Abstract] OR shift-work[Title/Abstract] OR shiftwork[Title/Abstract] OR shift worker[Title/Abstract] OR shift-worker[Title/Abstract] OR shift-workers[Title/Abstract] OR shiftworker[Title/Abstract] OR shift workers[Title/Abstract] OR shift work schedule[Title/Abstract] OR night shift work[Title/Abstract] OR evening shift work[Title/Abstract] OR rotating shift[Title/Abstract]) | 8790 |
|  | 3 | #1 AND #2 | 860 |
| Embase | 1 | ('cardiovascular diseases'/exp OR 'coronary artery disease'/exp) OR ('cardiovascular diseases':ti,ab,kw OR 'coronary disease ':ti,ab,kw OR 'coronary artery disease ':ti,ab,kw OR 'myocardial infarction':ti,ab,kw OR 'ischemic heart disease ':ti,ab,kw, 0R'coronaryheart disease':ti,ab,kw OR 'stroke':ti,ab,kw OR 'cerebral infarction ':ti,ab,kw OR 'cerebral hemorrhage ':ti,ab,kw OR 'cardiovascular morbidity ':ti,ab,kw OR 'cardiovascular mortality':ti,ab,kw) | 6262796 |
|  | 2 | 'shift schedule'/exp OR 'night work':ti,ab,kw OR 'shift work':ti,ab,kw OR 'shiftwork':ti,ab,kw OR 'shift worker':ti,ab,kw OR 'shift workers':ti,ab,kw OR 'night shift work':ti,ab,kw OR 'evening shift work':ti,ab,kw OR 'rotating shift ':ti,ab,kw | 12819 |
|  | 3 | #1 AND #2 | 1835 |
| Web of Science | 1 | TS= (Cardiovascular Diseases OR Coronary Disease OR Coronary Artery Disease OR Myocardial Infarction OR ischemic heart disease OR coronary heart disease OR Stroke OR cerebral infarction OR cerebral hemorrhage OR cardiovascular morbidity OR cardiovascular mortality)) OR AB=(Cardiovascular Diseases OR Coronary Disease OR Coronary Artery Disease OR Myocardial Infarction OR ischemic heart disease OR coronary heart disease OR Stroke OR cerebral infarction OR cerebral hemorrhage OR cardiovascular morbidity OR cardiovascular mortality) | 732766 |
|  | 2 | TS= (Shift Work Schedule OR shift work OR night work OR shift-work OR shiftwork OR shift worker OR shift-worker OR shift-workers OR shiftworker OR shift workers OR shift work schedule OR night shift work OR evening shift work OR rotating shift)) OR AB=(Shift Work Schedule OR shift work OR night work OR shift-work OR shiftwork OR shift worker OR shift-worker OR shift-workers OR shiftworker OR shift workers OR shift work schedule OR night shift work OR evening shift work OR rotating shift) | 96667 |
|  | 3 | #1 AND #2 | 1107 |
| Cochrane Library | 1 | (MeSH descriptor: [Cardiovascular Disease] explode all trees) OR (MeSH descriptor: [Coronary Artery Disease] explode all trees) OR (MeSH descriptor: [Stroke] explode all trees) OR (MeSH descriptor: [Myocardial Ischemia] explode all trees) OR (MeSH descriptor: [Coronary Disease] explode all trees) OR (Cardiovascular Disease):ti,ab,kw OR (Coronary Artery Disease):ti,ab,kw OR (Stroke):ti,ab,kw OR (Myocardial Ischemia):ti,ab,kw OR (Coronary Disease):ti,ab,kw) OR (ischemic heart disease):ti,ab,kw OR (coronary heart disease):ti,ab,kw OR (cardiovascular morbidity):ti,ab,kw OR (cardiovascular mortality):ti,ab,kw | 250384 |
|  | 2 | (MeSH descriptor: [Shift work schedule] explode all trees) OR (shift work schedule):ti,ab,kw OR (shift work):ti,ab,kw OR (night work):ti,ab,kw OR (shift-work):ti,ab,kw OR (shift worker):ti,ab,kw OR (shift-workers):ti,ab,kw OR (night shift work):ti,ab,kw OR (night evening work):ti,ab,kw OR (rotating shift):ti,ab,kw | 2712 |
|  | 3 | #1 AND #2 | 232 |
| CINAHL | 1 | (SU("Cardiovascular Disease" or "Coronary Artery Disease" or "Myocardial Ischemia" or "Coronary Disease" or "Stroke") OR TI("Cardiovascular Diseases" or "Coronary Disease" or "Coronary Artery Disease" or "Myocardial Infarction" or "ischemic heart disease" or "coronary heart disease" or "stroke" or "cerebral infarction" or "cerebral hemorrhage" or "cardiovascular morbidity" or "cardiovascular mortality") OR AB("Cardiovascular Diseases" or "Coronary Disease" or "Coronary Artery Disease" or "Myocardial Infarction" or "ischemic heart disease " or "coronary heart disease" or "stroke" or "cerebral infarction" or "cerebral hemorrhage" or "cardiovascular morbidity" or "cardiovascular mortality")) | 263089 |
|  | 2 | (SU("Shift Work Schedule") OR TI("shift work" or "night work" or "shift-work" or "shiftwork" or "shift worker" or "shift-worker" or "shift-workers" or "shift work schedule" or "night shift work" or "evening shift work" or "rotating shift") OR AB("shift work" or "night work" or "shift-work" or "shiftwork" or "shift worker" or "shift-worker" or "shift-workers" or "shift work schedule" or "night shift work" or "evening shift work" or "rotating shift") | 2924 |
|  | 3 | #1 AND #2 | 217 |
| Scoups | 1 | TITLE-ABS-KEY ( "Cardiovascular Diseases" OR "Coronary Disease" OR "Coronary Artery Disease" OR "Myocardial Infarction" OR "ischemic heart disease" OR "coronary heart disease" OR "Stroke" OR "cerebral infarction" OR "cerebral hemorrhage" OR "cardiovascular morbidity" OR "cardiovascular mortality" ) | 1754349 |
|  | 2 | TITLE-ABS-KEY ( "Shift Work Schedule" OR "shift work" OR "night work" OR "shift-work" OR "shiftwork" OR "shift worker" OR "shift-worker" OR "shift-workers" OR "shiftworker" OR "shift workers" OR "shift work schedule" OR "night shift work" OR "evening shift work" OR "rotating shift" ) | 16118 |
|  | 3 | #1 AND #2 | 1259 |

### Supplemental Table S2. NEWCASTLE - OTTAWA QUALITY ASSESSMENT SCALE (COHORT STUDIES)

| Author, year | Selection | | | | Comparability | Outcome | | | Overall  quality |
| --- | --- | --- | --- | --- | --- | --- | --- | --- | --- |
|  | Representative of cohort | Selection of cohort | Exposure ascertainment | No history of disease | Comparability of cohorts | Outcome assessment | Follow-up long enough (median ≥ 5 years) | Adequacy of follow up |  |
| Bigert et al, 2022 | 0 | 1 | 1 | 1 | 1 | 1 | 1 | 1 | 7 |
| Brown et al, 2009 | 1 | 1 | 1 | 1 | 2 | 1 | 1 | 1 | 9 |
| Bøggild et al, 1999, | 1 | 1 | 1 | 1 | 2 | 1 | 1 | 1 | 9 |
| Bunescu et al, 2024, | 1 | 1 | 1 | 1 | 1 | 1 | 0 | 1 | 7 |
| Chang et al, 2025 | 1 | 1 | 1 | 1 | 2 | 1 | 1 | 0 | 8 |
| Vetter et al, 2016 | 1 | 1 | 1 | 1 | 2 | 1 | 1 | 1 | 9 |
| Eng et al, 2022 | 1 | 1 | 1 | 1 | 1 | 1 | 1 | 1 | 8 |
| Ellingsen et al, 2007 | 1 | 1 | 1 | 1 | 1 | 1 | 1 | 0 | 7 |
| Fujino et al, 2006 | 1 | 1 | 1 | 1 | 2 | 1 | 1 | 0 | 8 |
| Gu et al, 2015 | 1 | 1 | 1 | 1 | 2 | 1 | 1 | 1 | 9 |
| Ho et al, 2021, | 1 | 1 | 1 | 1 | 2 | 1 | 1 | 0 | 8 |
| Hublin et al, 2010 | 1 | 1 | 1 | 1 | 2 | 1 | 1 | 0 | 8 |
| Jankowiak et al, 2024 | 1 | 1 | 1 | 1 | 2 | 1 | 1 | 1 | 9 |
| Jorgensen et al, 2017, | 1 | 1 | 1 | 1 | 2 | 1 | 1 | 0 | 8 |
| Kader et al, 2022 | 1 | 1 | 1 | 1 | 1 | 1 | 1 | 0 | 7 |
| Karlsson et al, 2005, | 1 | 1 | 1 | 1 | 1 | 1 | 1 | 1 | 8 |
| Li et al, 2021 | 0 | 1 | 1 | 1 | 2 | 1 | 1 | 1 | 8 |
| Larsen et al, 2019 | 1 | 1 | 1 | 1 | 2 | 1 | 1 | 1 | 9 |
| Tenkanen et al, 1997 | 1 | 1 | 1 | 1 | 2 | 1 | 1 | 1 | 9 |
| Vestergaard et al, 2023 | 1 | 1 | 1 | 1 | 2 | 1 | 1 | 1 | 9 |
| Wang et al, 2021 | 1 | 1 | 0 | 1 | 2 | 1 | 1 | 1 | 8 |
| Yadegarfar & McNamee, 2007 | 1 | 1 | 1 | 1 | 2 | 1 | 1 | 0 | 8 |
| Yong M et al, 2014 | 1 | 1 | 1 | 1 | 2 | 1 | 1 | 1 | 9 |

|  | Reviewer 2 (7★) | Reviewer 2 (8★) | Reviewer 2 (9★) | Total |
| --- | --- | --- | --- | --- |
| Reviewer 1 (7★) | 4 | 0 | 0 | 4 |
| Reviewer 1 (8★) | 0 | 11 | 0 | 11 |
| Reviewer 1 (9★) | 0 | 2 | 6 | 8 |
| Total | 4 | 13 | 6 | 23 |

Kappa score: 0.857 (0.70-1.00)

**1.2.3 Supplemental Table S3:** Evaluation of quality of pooled evidence using the Grading of Recommendations Assessment, Development and Evaluation (GRADE) framework

| Outcomes | Pooled outcomes | No. of patients (no. of included studies) | Statistical heterogeneity | Quality of evidence (GRADE) |
| --- | --- | --- | --- | --- |
| Total CVD incidence | 1.13(1.10 1.16) | 2,891,280  (15 studies) | I^2^=37.17%  *P=*0.07 | （⨁◯◯◯）^a^ |
| Total CVD morbidity | 1.27(1.19 1.36) | 638,255  (9 studies) | I^2^=13.51%  *P=*0.32 | （⨁⨁◯◯） |
| CHD incidence | 1.22(1.16 1.28) | 753,016  (6 studies) | I^2^=11.55%  *P=*0.34 | （⨁◯◯◯）^c^ |
| CHD morbidity | 1.22(1.10 1.36) | 214,742  (3 studies) | I^2^=0  *P=*0.74 | （⨁◯◯◯）^c^ |
| IHD incidence | 1.09(1.05 1.14) | 2,014,846  (5 studies) | I^2^=0  *P=*0.46 | （⨁◯◯◯）^c^ |
| IHD morbidity | 1.39(1.06 1.84) | 68,077  (4 studies) | I^2^=39.57%  *P=*0.17 | （⨁◯◯◯）^c^ |
| Stroke incidence | 1.06(0.95 1.18) | 632,886  (4 studies) | I^2^=16.38  *P=*0.31 | （⨁◯◯◯）^c, d^ |
| Stroke morbidity | 1.49(1.04 2.12) | 23,457  (2 studies) | I^2^=0  *P=*0.94 | （⨁◯◯◯）^b, c^ |

^a^ Downgraded by one level for the possibility of publication bias

^b^ Downgraded by one level for imprecision (total number of studies is less than 3)

^c^ Downgraded by one level for the possibility of publication bias which could not be evaluated due to insufficient studies.

^d^ Downgraded by one level for the 95% confidence interval includes meaningless effect values.

*No studies were downgraded for risk of bias since all studies ≥7 based on NOS.
